# Congenital cataract and congenital glaucoma in Marshall-Smith syndrome

**DOI:** 10.11604/pamj.2021.40.147.30762

**Published:** 2021-11-10

**Authors:** Jihene Sayadi, Ines Malek

**Affiliations:** 1 Department A, Hedi Rais Institute of Ophthalmology, Tunis El-Manar University, Tunis, Tunisia

**Keywords:** Marshall-Smith syndrome, congenital cataract, congenital glaucoma

## Image in medicine

Marshall-Smith syndrome (MSS) is an ultra rare congenital condition (prevalence <1/1.000.000) caused by de novo mutations involving the gene nuclear factor I/X. It is characterized by increased bone age, respiratory disorders, facial abnormalities and failure to thrive. We present a 22-day-old infant referred to our care centre for large bulging eyes and dysmorphism including prominent eyes, bilateral proptosis, depressed nasal bridge, anteverted nares, micrognathism, prominent forehead and hypertrichosis. Fiberoptic bronchoscopy concluded to severe laryngomalacia. On ophthalmic examination, the corneal diameter was 13.5 mm in the right eye (RE) and 14 mm in the left eye (LE). The intra-ocular pressure was 29 mmHg in the RE and 36 mmHg in the LE. Biomicroscopy showed severe corneal edema in both eyes. Corneal scarring secondary to hydrops has been noted in the LE. Bilateral total cataract precluded fundus examination. Ultrasound B-mode was unremarkable in both eyes. The patient passed away from respiratory compromise at the age of 29 days. Genetic testing has not been performed. However, facial features, the course of respiratory difficulty and ocular involvement were highly suggestive of the diagnosis of MSS.

**Figure 1:**
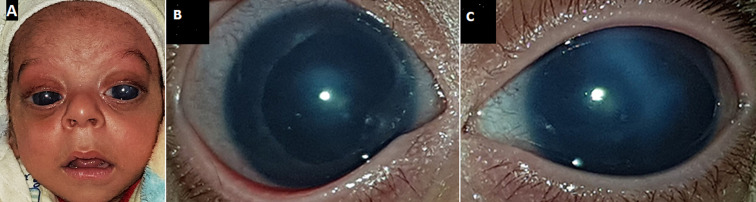
A) face photography of an infant with Marshall Smith syndrome showing signs of craniofacial dysmorphism including prominent eyes, bilateral proptosis, depressed nasal bridge, anteverted nares, micrognathism, prominent forehead and hypertrichosis; (B,C) anterior segment photography showing severe corneal edema and bilateral total cataract in both eyes; C) corneal scarring secondary to hydrops in the left eye

